# Balance control impairments in Fabry disease

**DOI:** 10.3389/fneur.2022.856946

**Published:** 2022-09-30

**Authors:** Laetitia Peultier-Celli, Roland Jaussaud, Pierre Kaminsky, Joëlle Deibener-Kaminsky, François Feillet, Philippe Perrin

**Affiliations:** ^1^EA 3450 DevAH - Development, Adaptation and Handicap, Faculty of Medicine, University of Lorraine, Vandoeuvre-lès-Nancy, France; ^2^Laboratory for the Analysis of Posture, Equilibrium and Motor Function (LAPEM), University Hospital of Nancy, Vandoeuvre-lès-Nancy, France; ^3^Department of Internal Medicine and Clinical Immunology, Vandoeuvre-lès-Nancy, France; ^4^Reference Centre for Inborn Errors of Metabolism, Children Hospital, University Hospital of Nancy, Vandoeuvre-lès-Nancy, France; ^5^Department of Pediatric Oto-Rhino-Laryngology, University Hospital of Nancy, Vandoeuvre-lès-Nancy, France

**Keywords:** Fabry disease, postural control, posturography, cochleo-vestibular disorders, rehabilitation

## Abstract

**Background:**

Fabry disease (FD) is a rare inherited lysosomal storage disorder caused by the deficiency of the enzyme alpha-galactosidase A. This deficiency leads to an accumulation of glycosphingolipids leading to progressive and multisystemic disease, including renal, cardiac, and neurological damages. FD may also have neuro-otological and visual impairments, which can generate postural control alterations, inner ear, and vision being involved in this function. This study aimed to evaluate the impact of FD on postural control.

**Methods:**

In total, fourteen adult patients (8 men/6 women, mean age = 37.6 ± 11.4 years) and two children (mean age = 11 years) with FD and 19 healthy adults (12 men/7 women, mean age = 36.5 ± 16.9 years) and two healthy children (mean age = 10.5 years) took part in this study. Postural control was evaluated by a sensory organization test combining three visual situations (eyes open, eyes closed, and sway referenced visual surround motion) with two platform situations (stable platform and sway referenced platform motion), aiming to calculate a composite equilibrium score (CES), a high score being representative of good postural control. Somatosensory (R^SOM^), visual (R^VIS^), and vestibular (R^VEST^) contributions to postural control were calculated, a low score reflecting a poor use of the indicated sensory input.

**Results:**

The CES was lower in adult patients with FD compared with the healthy subjects (*p* < 0.001). R^VIS^ (*p* = 0.001) and R^VEST^ (*p* = 0.003) were lower in patients with FD compared with the control group, whereas no difference in R^SOM^ was observed.

**Conclusion:**

Inner ear and visual pathologies associated with the central nervous system impairments are factors of postural control impairments. Physical activities, which can also be rehabilitative, by maintaining or increasing the weight of proprioception, may help diminish dependency on altered sensorial inputs.

## Introduction

Fabry disease (FD) (OMIM 301500) is an X-linked recessive inborn error of glycosphingolipid metabolism due to the deficient activity of the lysosomal enzyme alpha-galactosidase A (EC 3.2.1.22). The deficiency of alpha-galactosidase A leads to the storage of neutral glycosphingolipids, particularly, globotriaosylceramide and galactosylceramide, in many tissues and cell types (1). The accumulation of its substrate, the globotriaosylceramide (GL-3) leads to cellular dysfunction which might in turn trigger inflammation or fibrosis or both and results in a complex, and heterogeneous disease (2). The incidence of FD, is estimated between 1/35,000 and 1/476,000 births (3, 4). Both hemizygous males and heterozygous females can be affected. Phenotypic expression in heterozygous female depends on random X inactivation and male patients usually develop a more severe form of the disease with an earlier age at onset than female patients. The manifestations of FD are progressive and multisystemic. FD induces a progressive accumulation of GL-3 in the lysosomes of endothelial, perithelial, smooth–muscle cells of blood vessels, ganglion cells, and in many cell types in the heart, kidneys, eyes, and most other tissues (5, 6). Neurological (cerebrovascular and acroparesthesia), renal, cardiac, dermatological (angiokeratoma and hypohidrosis) involvements and corneal abnormality are the major clinical manifestations in patients with the classic phenotype (2). Vascular involvement contributes to the central nervous system abnormalities (7) and vascular ischemia and lipid deposition in the perineurium may cause the peripheral nerve conduction abnormalities seen in FD (8). Patients with FD suffer from sensorineural hearing loss, with both progressive hearing impairment (microvascular mechanism) and sudden deafness (macrovascular mechanism). These patients also present peripheral vestibular deficits with dizziness and vertigo. Vascular damage seems to be involved in the pathophysiologic mechanisms of cochleo-vestibular disorders (9). A correlation of neuropathic and vascular damage with hearing loss was found in men in whom residual alpha-galactosidase A activity appears to have a protective effect against hearing loss (10). Progressive vestibular loss was found in 80% of men and 77% of women when assessed with head impulse testing (11).

Postural control, which is an inner part of many ordinary activities, is a complex sensorimotor function that requires central integration of visual, vestibular, and proprioceptive/somatosensory systems. Integration of these three inputs generates a context-specific motor response, which leads to stabilization of gaze and antigravity posture (12–15). Visual and inner ear inputs, which can be affected by FD, contribute to the postural control.

Aging and a sedentary lifestyle are accompanied by a reduction in muscle mass and strength, which may be prevented or delayed by the practice of physical and sporting activities. Becoming skilled in the sporting activities helps to improve postural performance and as a consequence, reduce the number of falls (16).

To our knowledge, only one study included postural control evaluation in FD. This study, performed in eight patients, showed normal results in all the cases except for one whose postural control was abnormal with low scores in composite and vestibular component analysis (10). Our study aimed to evaluate the impact of FD on the postural control in treated and untreated patients.

## Methods

### Patients and controls

A case control study was conducted in the Nancy University Hospital (France) on 14 adult patients (8 men/6 women, mean age = 37.62 ± 11.43 years, ranging from 18 to 60 years) and two children (mean age = 11.01 ± 2.54 years) with FD issued from 6 families. Adult patients are those followed by the Department of Internal Medicine and Clinical Immunology and the children by the Reference Center for Inborn Errors of Metabolism, Children Hospital, University Hospital of Nancy.

A control group of 19 healthy adult volunteers (12 men and 5 women, mean age = 36.51 ± 16.99 years ranging from 21 to 72) and two children (mean age = 10.5 years ± 0.79) with no pathology took part in this study.

All the patients were included after neuro-otological examination, and the history of FD was recorded for each patient.

The history of FD was recorded for each patient and a clinical neuro-otological assessment aimed at detecting and discriminating central and vestibular signs, identifying segmental or axial deviations, and ruling out confounding associated factors. Concerning the vestibular syndrome, the consequences of a vestibular lesion were appreciated by evaluating the vestibulo-ocular pathway (presence of nystagmus) and the vestibulo-spinal pathway (which lesion can produce instability).

When abnormalities were observed at the clinical neurootological examination (including videonystagmoscopic head-shaking test and head impulse test) or if vertigo were reported, a more complete assessment was performed (caloric test, rotatory test, and skull-vibration-induced-nystagmus test), adapted to the age, the associated pathologies, and the side effects of the treatment of these pathologies.

This work has been conducted in accordance with The Code of Ethics of the World Medical Association (Declaration of Helsinki) for experiments involving humans and conducted in soundproof rooms for balance control recordings (Agence Régionale de Santé de Lorraine agreement for research). All the participants gave their informed consent prior to the clinical evaluation. For children, the parents and the participant gave their consent to participate.

### Posturographic analysis

**Figure 1:**
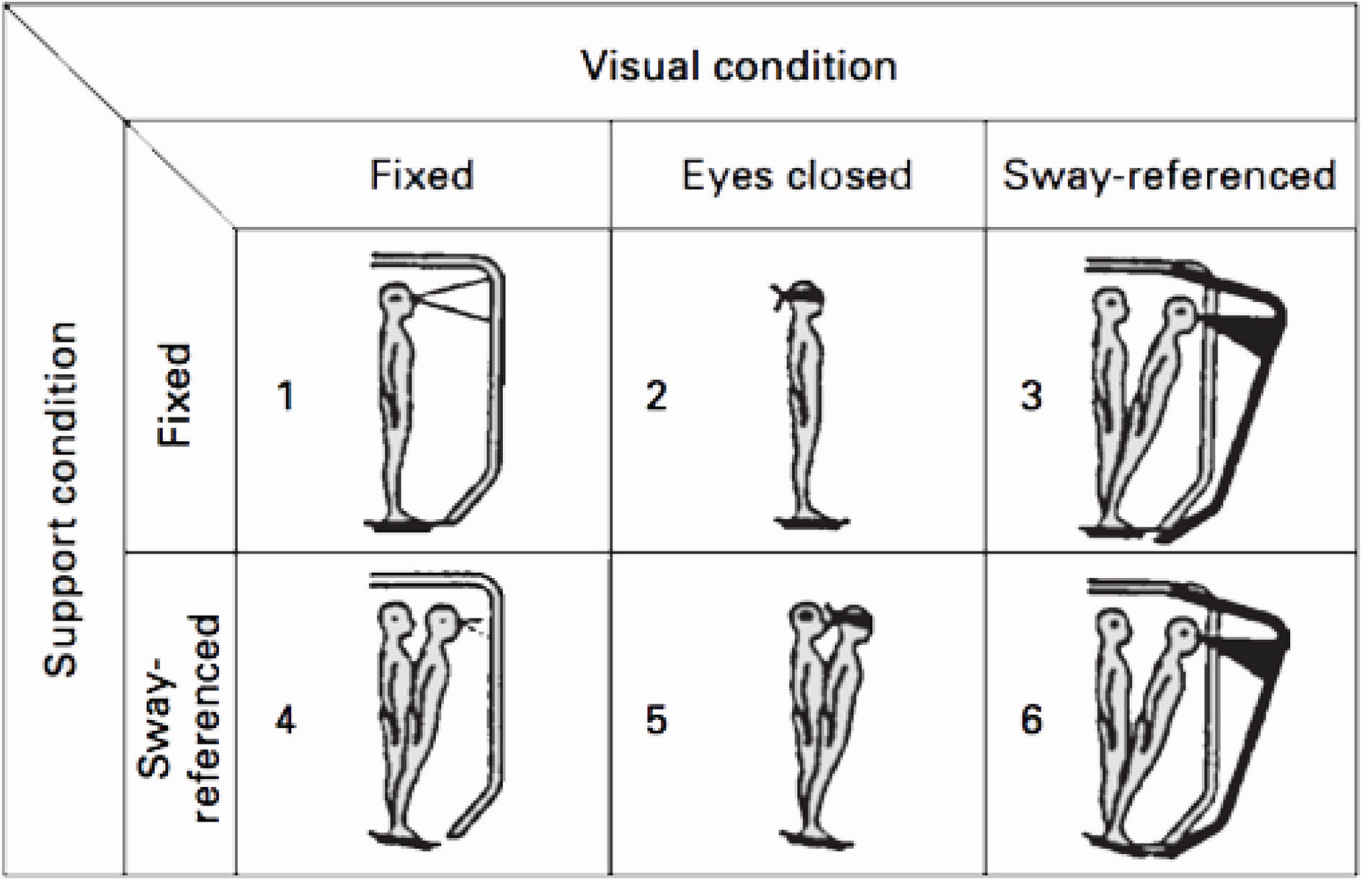
The six conditions of the SOT (EquiTest, NeuroCom International Inc., Clackamas, OR, USA). Conditions 1–3 were performed on a fixed platform with eyes open, eyes closed and vision sway-referenced. Conditions 4–6 were performed on a sway-referenced platform (somatosensory input inaccurate) with eyes open, eyes closed and vision sway-referenced (visual input inaccurate).

The Sensory Organization Test (SOT) was performed on an EquiTest computerized dynamic posturography platform (Neurocom, Clackamas, OR, USA). For the test, the subjects were requested to stand upright and barefoot on the platform, remaining as stable as possible, breathing normally, and with their arms at their sides, and were instructed to look straight ahead at a picture located on the visual surround. The SOT evaluates the patient's ability to make effective use of visual, vestibular, and somatosensory inputs separately and to suppress sensory information that is inappropriate. To give inadequate information, somatosensory and visual cues are disrupted by using a technique commonly referred to as sway-referenced. This technique involves tilting the support surface and/or the visual surround to directly follow the anterior–posterior sways of the subject's center of gravity (CoG) (17) (Figure 1). The SOT comprised six conditions (Table 1). The first two conditions provide a basic measurement of the subject's stability. The support is fixed and the subject's eyes are open (condition 1) or closed (condition 2). In condition 3, the support surface remains fixed while the subject stands, eyes open, within a sway-referenced visual surround. For conditions 4–6, somatosensory information is systematically disrupted (sway-referenced) and vision is fixed (condition 4), absent (condition 5), and sway-referenced (condition 6), respectively. In conditions 3 and 4, a sensory conflict is induced, but relatively easy to solve according to a ratio between the number of disrupted information and the number of reliable information (one disrupted cue for two reliable cues). The sensory conflict is more difficult to solve in conditions 5 (one disrupted cue for one reliable cue and vision being absent) and 6 (two disrupted cues for one reliable cue). For each condition, the subject maintains an upright stance during three 20 s trials with as little sway as possible and without moving the feet. When the subject required the assistance of the harness or took a step, the test was rated a fall. An Equilibrium Score (ES) was calculated by comparing the patient's anterior–posterior sway during each 20 s SOT trial to the maximal theoretical sway limits of stability. The theoretical limit of stability is based on the individual's height and size of the base of support (8.5° anteriorly and 4.0° posteriorly).

**Table 1:** Sensory organization test.

**Conditions**	**Situation**	**Sensory consequences**
Condition 1 (C1)	Eyes open, fixed support	-
Condition 2 (C2)	Eyes closed, fixed support	Vision absent
Condition 3 (C3)	SR surround, fixed support	Altered vision
Condition 4 (C4)	Eyes open, SR support	Altered proprioception
Condition 5 (C5)	Eyes closed, SR support	Vision absent, altered proprioception
Condition 6 (C6)	SR surround, SR support	Altered vision and proprioception
**Ratios**		**Significance**
Somatosensory (R^SOM^)	C2/C1	Question: does sway increase when visual cues are removed? Low scores: poor use of somatosensory references
Visual (R^VIS^)	C4/C1	Question: does sway increase when somatosensory cues inaccurate? Low scores: poor use of visual references
Vestibular (R^VEST^)	C5/C1	Question: does sway increase when visual cues are removed and somatosensory cues are inaccurate? Low scores: poor use of vestibular cues or vestibular cues unavailable

Determination of the six conditions and significance of sensory ratios (17–19).

The composite equilibrium score (CES) was calculated by adding the average scores from conditions C1 + C2 + 3 x C3 + 3 x C4+ 3 x C5+ 3 x C6 / 14.

SR, sway-referenced.

A composite equilibrium score (CES) was calculated by adding the average scores from conditions 1 and 2 and the ES from each trial of sensory conditions 3, 4, 5, and 6, and finally dividing that sum by the total number of trials (Table 1).

Lower sways lead to a higher CES, indicating a better balance control performance (a score of 100 represents no sway, while 0 indicates sway that exceeds the limit of stability, resulting in a fall).

### Statistics

Results between the two adult groups are presented as mean ± SD. Comparison of qualitative variables used the Fisher's exact test while that of quantitative variables was performed using the non-parametric Mann Whitney *U*-test. Multivariate analysis used a multiple logistic regression model. A *p*-value lower than 0.05 was considered as significant.

## Results

No significant differences were observed between the two groups for age, height, weight, BMI, and sex. Acroparesthesia were present in 64.3% (9 patients), 57% of them (8 patients) suffered from dizziness (accompanied by orthostatic hypotension for 28.6% [3 patients, one of them related to side effects of antihypertensive treatment (severe HCM)], 42.9% of them suffered of tinnitus (6 patients), 85.7% have a hearing loss (57% have a slight hearing loss (8/14 patients), 28.6% have a moderate hearing loss (4/14 patients), and 7.1% have an unilateral sudden hearing loss (1/14 patient); 64.3% of adult patients (9/14) were treated by enzyme replacement therapy (ERT) (Table 2). In the adult patients, 7/8 men and 5/6 women had hearing loss.

Table 2Fabry disease patients characteristics.

**Table:** 

**Part 1: Clinical characteristics**												
**Patients**	**No**	**Age range (y)**	**Same family members**	**Hearing loss**	**Tinnitus**	**Vertigo/ Dizziness**	**Vision**	**Somesthesia**	**CNS**	**Gaze stabilization**	**Postural control**	**Organ failure**
Family 1	1	15–19	2, 3, 4	Slight	+		Myopia	Acroparesthesia		Bilateral end-point nystagmus		
	2	20–24	1, 3, 4	Slight			Astigmatism	Acroparesthesia				
	3	25–29	1, 2, 4	Normal		+	CV, astigmatism, hypermetropia	Acroparesthesia		Bilateral end-point nystagmus		
	4	45–49	1, 2, 3	Moderate		+ orthostatic hypotension	Astigmatism, myopia		Cephalalgia (visual)	Vergence deficit		
Family 2	5	25–29		Slight				Acroparesthesia		Vergence deficit		
	6	30–34	7	Normal			Hypermetropia	Acroparesthesia	MRI periventricular hypersignals			Nephrotic syndrom
	7	60–64	6	Moderate	+	+ orthostatic hypotension				Bilateral end-point nystagmus		Severe HCM
Family 3	8	10–14	9, 10, 11, 12	Slight							Instability	
	9	5–9	8, 10, 11, 12	Normal								
	10	35–39	8, 9, 11, 12	Unilateral sudden hearing loss	+			Acroparesthesia	MRI periventricular hypersignals	Vergence deficit, saccadic ocular pursuit		HCM
	11	35–39	8, 9, 10, 12	Slight	+	+		Acroparesthesia	Pituitary adenoma		Instability	
	12	40–44	8, 9, 10, 11	Slight	+	+ orthostatic hypotension		Acroparesthesia			Instability	Mild HCM
Family 4	13	35–39	14	Slight		+ (VVS, epilepsia)	Astigmatism, myopia		MRI periventricular hypersignals	Bilateral end-point nystagmus, vergence deficit	Instability	
	14	40–44	13	Slight		+			Migraine, darkness phobia		Instability	
Family 5	15	50–54		Moderate	+	+	CV, astigmatism, hypermetropia	Acroparesthesia		saccadic ocular pursuit		Moderate HCM
Family 6	16	40–44		Moderate			CV, astigmatism, hypermetropia		MRI periventricular hypersignals			ESRD

**Table:** 

**Part 2: Genetic mutations and treatments**								
**Families**	**No**	**Age range (y)**	**Mutation**	**Nucleotide aberration**	**Megalastat sensible**	**ERT**	**Treatment duration (y)**	**Other treatments**
1	1	15–19	IVS4-2 A>T	c.640-2A>T	−	AA	3	
	2	20–24	IVS4-2 A>T	c.640-2A>T	−	AA	3	
	3	25–29	IVS4-2 A>T	c.640-2A>T	−	AA	0.5	
	4	45–49	IVS4-2 A>T	c.640-2A>T	−	-	0	Anti AHT
2	5	25–29	p.A143T	c.427G > A	+	-	0	
	6	30–34	p.A143T	c.427G>A	+	AB	3	
	7	60–65	p.A143T	c.427G>A	+	AB	0.5	Anti vertiginous
3	8	10–14	p.M42R	c.125 T>G	+	-	0	
	9	5–9	p.M42R	c.125 T>G	+	-	0	
	10	35–39	p.M42R	c.125 T>G	+	AA	1	Anti AHT
	11	35–39	p.M42R	c.125 T>G	+	-	0	Vestibular Rehabilitation, anti vertiginous
	12	40–44	p.M42R	c.125 T>G	+	AA	1	
4	13	35–39	p.P205S	c.613 C>T	+	-	0	Antiepileptic
	14	40–44	p.P205S	c.613 C>T	+	-	0	Balance Rehabilitation
5	15	50–54	del 50 pb Ex7	del 50 pb Ex7	−	AA	5	
6	16	40–44	-	−	-	AA	4	

Anti AHT, anti-arterial hypertension; HCM, hypertrophic cardiomyopathy; CV, cornea verticillata; ESRD, End-stage renal disease; VVS, vasovagal syndrome; ERT, Enzyme replacement therapy; AA, AB, agalsidase alfa, beta; MRI, Magnetic Resonance imaging.

No genotype–phenotype correlation was found for hearing/balance disorders.

### Posturography analysis

**Figure 2:**
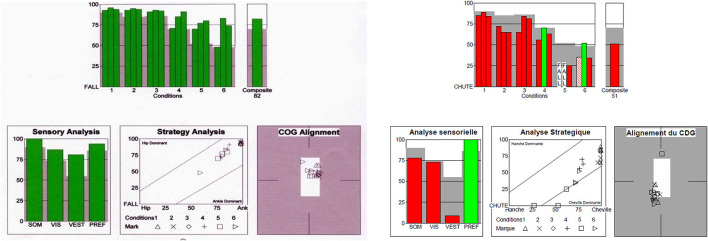
Sensory organization test. On the left: normal values of the Composite score (82) and of R^SOM^, R^VIS^, R^VEST^. Strategy analysis: ankle dominant, Center of gravity (COG) alignment: normal. On the right: Fabry disease patient (patient No 14): low Composite score (51), low values of R^SOM^ and R^VEST^. Strategy: ankle and hip dominant strategies and falls according to the condition. Center of gravity shifted to the backward position.

In adults, patients with FD showed significantly lower composite equilibrium score (CES) (73.1 ± 8.7 vs. 83.8 ± 5.3 (*p* < 0.001), visual ratio (R^VIS^) (0.831 ± 0.087 vs. 0.932 ± 0.047; *p* = 0.001) and vestibular ratio (R^VEST^) (0.534 ± 0.282 vs. 0.750 ± 0.097; *p* = 0.003) compared with the control group whereas no difference of somatosensory ratio (R^SOM^) (*p* = 0.182) were observed (Figure 2, Table 3). In the adult patients, postural control was impaired in 3/8 men and 5/8 women.

**Table 3:** Posturographic and vestibular results in the Fabry disease patient group.

**Patients**	**Vertigo**	**Composite score (CES)**	**Vestibular ratio (RVEST)**	**Visual ratio (RVIS)**	**Somatosensory ratio (RSOM)**	**Posturographic pattern**	**Vestibular examination**
1		77	0.71	0.79	0.99	RVIS slightly 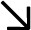	-
2		78	0.62	0.83	1.01	Normal	-
3	**+**	81	0.70	0.93	0.97	Normal	Nothing to notice
4	**+**	74	0.65	0.70	1.00	RVIS 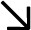	Interpretation disturbed by strabismus
5		82	0.68	0.89	0.99	Normal	-
6		85	0.83	0.98	0.96	Normal	-
7	**+**	83	0.72	0.95	0.98	Normal	Nothing to notice (orthostatic hypotension)
8		51	0.00	0.68	0.86	RVEST 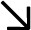 RVIS 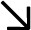 CES 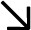	Left vestibular areflexia
9		72	0.59	0.84	0.99	Normal	-
10		76	0.65	0.77	0.96	RVIS 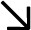	-
11	**+**	66	0.00	0.88	0.85	CES 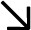 RVEST 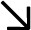	Bilateral vestibular areflexia
12	**+**	49	0.00	0.77	0.85	RVEST 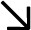 RVIS 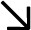 CES 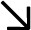	Left vestibular areflexia
13	**+**	67	0.52	0.75	0.94	CES 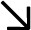 RVEST 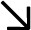 RVIS 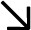	Interpretation disturbed by epilepsy treatment and vergence deficit
14	**+**	51	0.10	0.73	0.78	CES 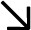 RVEST 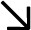 RSOM 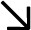	Bilateral vestibular hyporeflexia
15	**+**	72	0.58	0.79	0.96	RVEST 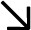 RVIS slightly 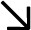	Vestibular central pathology Saccadic ocular pursuit
16		83	0.72	0.87	0.98	Normal	-

Enzyme replacement therapy did not influence postural measures. However, patients with postural control instability (4 patients) presented more frequently impaired CES (*p* < 0.001), R^SOM^ (*p* < 0.02), and R^VEST^ (*p* < 0.001) than the other patients and were more frequently women than men (*p* < 0.005). In the multivariate analysis, altered R^VEST^ remained the sole independent factor explaining abnormal postural control in patients with FD.

Postural control analysis was also performed on the other two younger patients with FD, one age range 10–14 y and one age range 5–9 y. Postural control was lower in the oldest child compared with the two healthy children while no difference was observed in the youngest one compared with the healthy children.

### Vestibular examination

Vertigo and/or R^VEST^ abnormalities were noted in patients 3, 4, 7, 8, 11, 12, 13, 14, and 15.

There were no abnormalities on complementary examinations in patients 3 and 7. The interpretation was disturbed in patient 4 by a strabismus. In patient 13, the results were disturbed by the side effects of the antiepileptic treatment. Unilateral vestibular areflexia was observed in patients 8 and 12 and bilateral vestibular hyporeflexia in patient 14, and areflexia in patient 11. In patient 15, eye pursuit was saccadic.

## Discussion

This study showed that balance control performance was lower in adult patients with FD patients compared with the healthy subjects. Inner ear and visual pathologies associated with the central nervous system impairments are the main factors of postural control impairments in these patients whereas no difference was observed between the two groups in the use of somesthetic input.

Fabry disease frequently leads to the inner ear dysfunctions, such as sensorineural hearing loss, sudden deafness, tinnitus, and dizziness or vertigo (20). Hearing loss in FD is due to the accumulation of GL-3 in the inner ear (21). In the mouse the loss of a-galactosidase A activity is genetically or biochemically buffered and not sufficient *per se* to cause an appreciable degree of hearing impairment (22). Although the alpha-galactosidase A deficient mice showed no clear hearing loss, GL-3 accumulation was demonstrated in the cochlea (21). The data demonstrate that in the mouse the loss of α-galactosidase A activity is genetically or biochemically buffered and not sufficient *per se* to cause an appreciable degree of hearing impairment.

Histopathologic evidence of glycosphingolipid accumulation in vascular endothelial and ganglion cells, and also atrophy of the stria and spiral ligament, might explain the otoneurologic symptoms (23).

To date, it is not known if ERT can reduce or prevent the cerebrovascular complications and hearing loss associated with FD, and it is unclear whether vertigo and tinnitus can be improved with ERT. It has been hypothesized that since migalastat is able to cross the blood–brain barrier, it might contribute in reducing the occurrence of cerebrovascular events (24).

Progressive sensorineural hearing loss can be stabilized, but not reversed (with or without treatment), but frequency of sudden hearing loss decreases during ERT compared with the frequencies observed in untreated patients (25). ERT does not appear to be a recognized therapy for sudden hearing loss. Therapeutic goals for hearing loss include stabilization of hearing loss, the possible use of hearing aids or cochlear implants to improve both hearing and patient quality of life (26).

No age effect was observed in the adult patients and one of both children (age range 10–14 y) with low posturographic performances do not present other clinical signs than postural instability.

Genotype–phenotype correlation remains controversial in FD and there is no specific data about hearing/balance disorders in term of genotype–phenotype correlation. In FD, more than 1,000 variants have been described and this high number underly the phenotype heterogeneity observed in this disease. Most of variants are “private” and confined to individual pedigrees with possible variability in phenotypic expression due to phenotype-modifying factors: i.e., genetic background, epigenetics, and environmental conditions (27, 28). One recent paper described the genotype–phenotype correlation in term of event-free but not in function of each possible clinical event, and in particular, there is no specific data about hearing/balance disorders in these patients (29). Moreover, in women patients, the phenotype depends of the genotype but also (and mainly) of the X-chromosome inactivation (30).

Patients with FD respond inappropriately to conflicting or inaccurate sensorial inputs. Vestibular alteration may explain a lower inner ear contribution in postural control (R^VEST^) with lower values in postural control (CES) in patients with FD compared with the healthy subjects.

Concerning the 4 patients with vestibular hypo or areflexia, all 4 had a slight hearing loss (16 to 20 dB). The combination of vertigo and hearing loss may point to the inner ear as the origin of the disorder because of anatomic proximity of the vestibular and cochlear structures, whereas a lack of correlation between vestibular and cochlear disorders may suggest different pathophysiological mechanisms for these two structures.

Among the four patients with the vestibular disorders, two were unilateral and two were bilateral and symmetrical. Slow progressive vestibular damage, through the development of vestibular compensation mechanisms involving effective use of alternative sensory inputs, may allow the patient to experience few symptoms.

Vergence disorders and saccadic ocular pursuit contribute also to a lower postural control. Strabismus may interfere with pursuit and visual fixation and be accompanied by ophthalmic nystagmus. This oculomotor disturbance may suggest central vestibular damage. Because of the vestibuloplegic effect (antiepileptics) or the induction of nystagmus or abnormal eye movements (antiepileptics and anti-inflammatory drugs) of some of the prescribed drugs, vestibulo-ocular reflex analyses were difficult to interpret. Dizziness could be induced by inner ear toxicity (bilateral toxicity) or by the central damage.

Corneal abnormalities (cornea verticillata), neither responsible for changes in visual acuity nor causing complaints, did not decrease the weight of visual input in postural control. None of the patients with FD in our study had a visual dependency or preference.

Acroparesthesia did not cause a decrease in somesthetic afference weight.

The presence of both a decrease in visual and vestibular afference may reflect a central integration defect.

Usher syndrome, an autosomal recessive disorder, is characterized by congenital hearing loss combined with retinitis pigmentosa, and in some cases, vestibular areflexia, leading to postural disability, that may have similar posturographic results to those of the FD (31).

In patients with FD, recovery in treated patients, i.e., *a priori* the most severe forms, would be in favor of damage to peripheral structures and a potentially curative action of the treatment. In our study, in adults, the composite score (CES) was better in treated patients than in untreated patients (76 vs. 69), as were R^SOM^ (0.96 vs. 0.92), R^VIS^ (0.86 vs. 0.79), and R^VEST^ (0.61 vs. 0.43), the results favoring peripheral involvement not being significant, however, due to the small size of the population.

Since the patients had comparable characteristics to the controls (in particular, age, sex, and body mass index), it was considered that if the patients showed statistically lower values than the control group, this could probably be due to their pathology. Nevertheless, based only on the average of the CES, patients with FD have values of balance (measured by dynamic posturography) close to the norms of the device, although worse than healthy individuals of the same age.

Enzyme replacement therapy treatment does not cross the blood–brain barrier and, therefore, cannot have any positive effect on the central component of the disorder.

The efficacy of the treatment would be more in favor of an effect of ERT on microvascular involvement of FD. This improvement in microvascularization could stabilize the inner ear functioning, which would require long-term follow-up to be confirmed.

This study has several limitations. The limited sample size of the population and in particular of the children should be highlighted, which limits in particular the study of the difference in symptomatology between men and women and of the impact of the type of treatment on balance and hearing. The postural control will need to be considered in the evaluation of current and future treatments for FD, whether migalastat or gene therapy. According to Palla et al., age correlated with auditory and vestibular impairments (11). The evaluation of the type of balance control recovery of vestibular disorders (i.e., recovery by compensation involving effective use of alternative sensory inputs or recovery by restoration) could require a longitudinal follow-up by vestibular and posturographic investigations, in situations of sensory conflict.

We emphasize the importance of multisensory evaluation in these patients to guide development of personalized visuo-vestibular rehabilitation techniques.

Otherwise, as we age, balance control becomes more and more dependent on vision, while this input becomes increasingly less performant. Training allows sportsmen to acquire new balance control abilities, which may differ according to the discipline practiced. Physical activity may help diminish this visual dependency by maintaining or increasing weight of proprioceptive input (32).

Physical activity practice (e.g., tai-chi, yoga) or physiotherapy can be useful to prevent balance control impairments. The beneficial effect of this training may then be transferred to daily activities.

## Conclusion

These data suggest that understanding of specific balance control impairments in FD could contribute to propose better balance-oriented rehabilitation programs with the particular attempt of preventing falls. The multisensory evaluation of postural control helps determine the adapted modalities of visuo-vestibular rehabilitation and to evaluate in a quantifiable way its effect to improve postural stability and quality of life.

## Data availability statement

The original contributions presented in the study are included in the article/supplementary materials. Requests may be directed to the following clinicians (MD): RJ: R.JAUSSAUD@chru-nancy.fr, JD-K: j.deibener@chru-nancy.fr, FF: f.feillet@chru-nancy.fr, and PP: philippe.perrin@univ-lorraine.fr. Further inquiries can be directed to the corresponding author.

## Ethics statement

Ethical review and approval was not required for the study on human participants in accordance with the local legislation and institutional requirements. Written informed consent to participate in this study was provided by the patients/participants or patients/participants' legal guardian/next of kin.

## Author contributions

LP-C realized posturography recordings. PK, FF, RJ, and JD-K carried out the internal medicine assessment and therapeutic management of the FD. PP managed the neuro-otological assessments. All authors contribute to the study concept and design, and contribute to the writing of the manuscript.

## Conflict of interest

The authors declare that the research was conducted in the absence of any commercial or financial relationships that could be construed as a potential conflict of interest.

## Publisher's note

All claims expressed in this article are solely those of the authors and do not necessarily represent those of their affiliated organizations, or those of the publisher, the editors and the reviewers. Any product that may be evaluated in this article, or claim that may be made by its manufacturer, is not guaranteed or endorsed by the publisher.
